# Significantly Elevated Liver Alkaline Phosphatase in Congestive Heart Failure

**DOI:** 10.14740/gr600w

**Published:** 2014-05-02

**Authors:** Leonid Shamban, Brijesh Patel, Michael Williams

**Affiliations:** a Department of Internal Medicine, Providence Hospital and Medical Center, 16001 W Nile Mile Road, Southfield, MI 48075, USA

**Keywords:** Alkaline phosphatase, Congestive hepatopathy, Heart failure

## Abstract

Congestive hepatopathy can have a mildly elevated liver profile, which should normalize with appropriate therapy. Liver specific alkaline phosphatase (ALP) in decompensated heart failure (HF) can be mildly elevated. The levels exceeding beyond the expected rise should be a concern and lead to further investigation. The literature reports insubstantial number of cases regarding significantly elevated levels of ALP and congestive hepatopathy. We report a case of a 45-year-old female with known history of severe cardiomyopathy that had persistently elevated levels of ALP. The extensive workup was negative for any specific pathology. The liver biopsy was consistent with congestive hepatopathy. The patient’s ALP levels decreased with aggressive diuretic therapy but still remained elevated.

## Introduction

Alkaline phosphatase (ALP) is commonly monitored in clinical practice and can be elevated in various hepatobilliary disease processes. ALP is a heterogenous group of enzymes that catalyze phosphate esters into an organic compound and inorganic phosphate. In humans, it is found in liver, bones, intestine, placenta, kidneys and leukocytes [1]. The liver and bone isoenzymes are the most clinically relevant due to the disproportionate synthesis from these organs. Hepatic ALP is concentrated near the biliary canalicular membrane of the hepatocyte [2], and in pathologic conditions, the respective isozymes can be elevated which helps to localize the source of the disease. Concomitant elevations of alanine aminotransferase (ALT), aspartate aminotransferase (AST) and gamma-glutamyltransferase (GGT) indicate the elevation of ALP is from a hepatic source [2]. The common causes of elevated ALP include primary sclerosing cholangitis, primary billiary cirrhosis, billiary obstruction, infiltrative diseases, Budd-Chiari obstruction and congestive hepatopathy [3]. In most cases, the ALP levels are mild or moderately elevated in these conditions. The persistently elevated levels of ALP are not only perplexing but often lead to further workup in order to determine the source. Typically very high levels of liver ALP are observed in patients with neoplasms of the liver or billiary tract, severe sepsis, Paget’s disease and AIDS [4, 5], but reports on high level of ALP in association with heart failure (HF) are lacking. Herein, we report a case of an elevated level of ALP that is out of proportion for a patient with acute on chronic systolic HF.

## Case Report

A 45-year-old African American female with history of severe non-ischemic cardiomyopathy with an ejection fraction of 20% and poorly controlled diabetes mellitus type 2, presented to the hospital with difficulty in breathing that had been ongoing for several days. She also complained of orthopnea, lower extremity swelling and a weight gain of least 30 lb. Her initial heart rate was 102 beats per minute, blood pressure of 93/73 mm Hg (with her baseline systolic blood pressure, 90 - 100 mm Hg), respiratory rate of 18 breaths per minute and a temperature of 97.4 °F. The physical exam revealed bibasilar crackles, a regular rate and rhythm, loud P2, jugular venous distention, moderate abdominal distension with significant subcutaneous swelling and a +2 pitting edema of the lower extremities.

Her laboratory workup showed NT-proBNP 11,184 pg/mL (192 pg/mL upper limit of normal), troponin < 0.02 ng/dL (normal range: 0 - 0.10 ng/dL) and no electrolyte abnormalities. The electrocardiogram did not indicate any signs of ischemia or arrhythmia. Additional laboratory workup revealed AST 28 unit/L, ALT 21 unit/L, ALP 658 unit/L (129 unit/L upper limit of normal), total bilirubin of 1.0 mg/dL and an albumin of 2.2 g/dL (Table 1).

**Table 1:** Initial Laboratory Studies the Patient Presented With on Admission

Labs on admission	Patient’s value	Normal value
Prothromin time (PT)	14.2	10.0 - 13.5 s
INR	1.2	< 1.5
Activated partial thromboplastin time (aPTT)	31.6	25.0 - 36.0 s
Aspartate aminotransferase (AST)	28	10 - 35 units/L
Alanine aminotransferase (ALT)	21	10 - 35 units/L
Albumin	2.2	3.5 - 5.2 g/dL
Alkaline phosphatase	658	35 - 129 units/L
Liver alkaline phosphatase	95	44-84%
Bilirubin total (Bili T)	1	0.1 - 1.0 mg/dL
Erythrocyte sedimentation rate (ESR)	9	0 - 20 mm/h
pro-BNP	11,184	50 - 192 pg/mL
Hemoglobin A1C	> 17.4	4.8-5.9%

**Figure 1:**
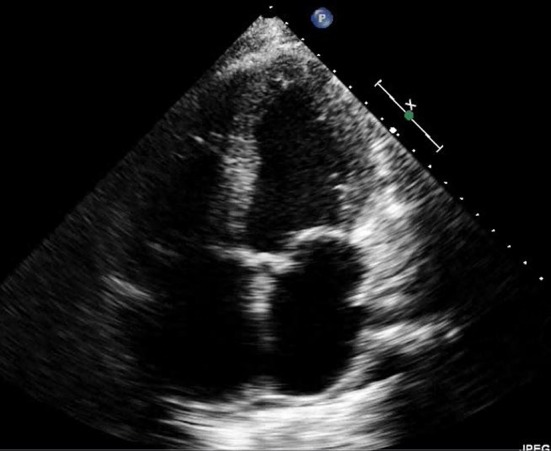
Transthoracic echocardiography (in end-systolic phase) shows an evidence of severe cardiomyopathy with ejection fraction < 20%.

Her coagulation panel and platelets were within normal limits. The transthoracic echocardiography showed severe cardiomyopathy with ejection fraction less than 20% (Fig. 1), and right ventricular, inferior vena caval dilatation. Based on the clinical picture, biventricular HF was diagnosed secondary to medication noncompliance, and the patient was treated with aggressive intravenous diuretics.

To investigate the marked elevation of ALP, initially the patient’s medications were reviewed, with no history of over-the-counter or herbal medications to explain such a rise. Therefore, an additional workup was then pursued (Table 2).

**Table 2:** Serological Studies That Were Checked to Evaluate the Elevated ALP

Serological evaluation of ALP	Patient’s value	Normal value
Thyroid stimulating hormone (TSH)	2.42	0.27 - 4.20 mcIU/mL
Iron level	52	37 - 145 mcg/dL
Total iron binding capacity (TIBC)	281	228 - 428 mcg/dL
Iron saturation	19	20-55%
Ferritin	47	13 - 150 ng/mL
Ceruloplasmin	43	16 - 45 mg/dL
Anti-nuclear antibody	Negative	Negative
Mitochondrial antibody	Nondetected	Nondetected
Smooth muscle antibody	Nondetected	Nondetected
Hepatitis A IgM	Nondetected	Nondetected
Hepatitis B core antibody IgM	Nondetected	Nondetected
Hepatitis B surface antigen	Nondetected	Nondetected
Hepatitis C antibody	Nondetected	Nondetected
Angiotensin converting enzyme	85	8 - 52 units/L
Alfa fetoprotein	1.1	0 - 13.0 ng/mL
Carcinoembryonic antigen (CEA)	4.7	< 5.0 ng/mL
Cancer antigen 19-9 (CA 19-9)	2	0.0 - 35.0 units/mL
Cytomegalovirus (CMV) antibody IgM	0.03	0 - 0.69 index
Cytomegalovirus (CMV) antibody IgG	0.2	0.0 - 0.8 AI
Herpes simplex virus (HSV) 1 antibody IgM	0.78	0.00 - 0.90 index
Herpes simplex virus (HSV) 1 antibody IgG	4.4	0.0 - 0.8 AI
Quantiferon TB-gold	Negative	Negative

**Figure 2:**
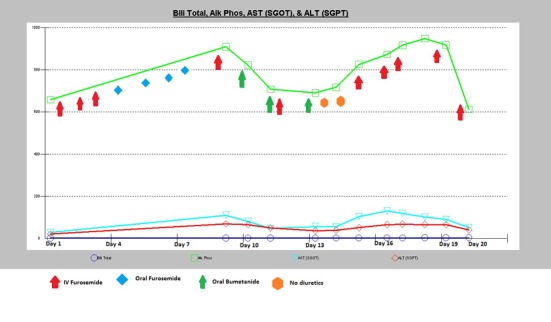
This graph illustrates how ALP levels responded to various types of diuretic therapies along with the other liver enzymes progressions; total bilirubin, AST and ALT levels.

Her ALP continued to trend upwards with mild elevation of transaminases and total bilirubin (Fig. 2).

**Figure 3:**
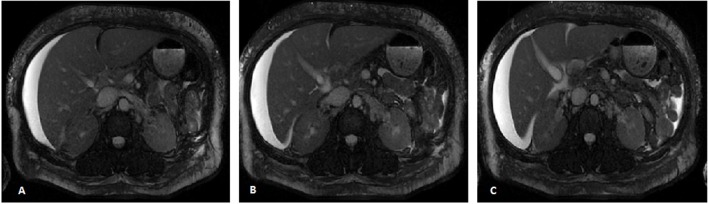
Three consecutive axial MR images, images A-C, of the abdomen demonstrating a normal appearing liver with moderate perihepatic ascites along with gallbladder wall thickening. Biliary dilatation is not demonstrated, with a normal pancreatic duct and a common bile duct measuring 3 mm.

An ultrasound of the gallbladder revealed calculi with sludge along with wall thickening but no biliary ductal dilatation. A cholescintigraphy scan demonstrated a normal ejection fraction of 45% with no evidence of cystic duct obstruction. A magnetic resonance cholangiopancreatography (MRCP) revealed pericholecystic fluid with gallbladder wall thickening without evidence of biliary obstruction or suggestive findings of primary sclerosing cholangitis (Fig. 3). A subsequent ultrasound with additive arterial and venous evaluation did not reveal findings of thrombosis.

Despite aggressive therapy, the patient had a positive fluid balance and started to develop signs of cardiorenal syndrome. She was evaluated by an HF specialist, and was deemed not be a candidate for left ventricular assist sevice or heart transplantation. The patient made an initial response to a furosemide drip with a negative fluid balance, and 48 h afterwards the patient was switched to oral loop diuretics. However, the subsequent day, the patient continued to retain fluid and ultrafiltration along with hemodialysis was considered. Her ALP continued to rise even with aggressive diuresis and stabilized at 948 unit/L with mild elevation of transaminases with AST 52 unit/L and ALT 40 unit/L. Her total bilirubin slightly increased to 2.2 mg/dL along with a mild increase in her prothrombin time to 15.5 s and INR of 1.3.

**Figure 4:**
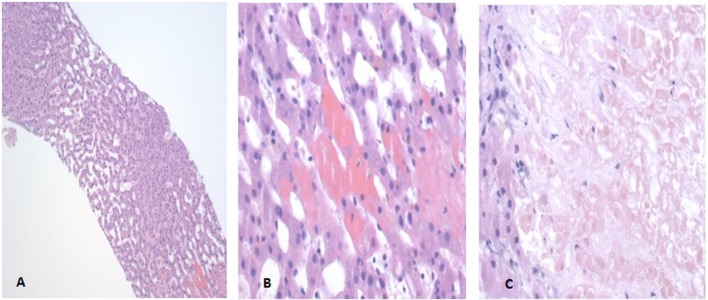
Liver Biopsy Pathology Slides. (A) The hematoxylin and eosin (H&E) stain at 100× depicting dilated sinusoids of the liver suggestive of right-sided heart failure. (B) The H&E stain at 400× depicting erythrocytes in hepatic plates suggestive of left-sided heart failure. (C) The H&E stain at 400× demonstrating biliary infarction consistent with mild biliary obstruction.

A CT guided random liver core biopsy was performed with four specimens obtained. The liver biopsy findings revealed venous congestion and mild biliary obstruction (Fig. 4). There was no evidence to suggest sarcoidosis, amyloidosis or lymphocytic malignancies. Unfortunately, later in the hospitalization the patient went into respiratory failure followed by cardiac arrest.

## Discussion

This case represents an atypical presentation of an elevated liver ALP in the presence of severe congestive HF. Congestive hepatopathy can have mildly elevated liver profile, which should normalize with appropriate therapy. Hepatic congestion may respond to diuresis, but in severe HF, augmentation of cardiac output may also be required to prevent further deterioration of liver function. In cases that are refractory, patients may undergo ultrafiltration to remove edema/ascites that is no longer responsive to diuretic therapy [6]. This notion is seen in our patient whose ALP started to improve when there was response to diuresis therapy (Fig. 2). However, as the diuresis waned ultrafiltration and hemodialysis was then considered.

Specific ALP in decompensated HF can be mildly elevated. Abnormalities in liver enzymes are not an uncommon finding in patients with HF [6]. Increased levels of AST and ALT in HF have been attributed to hepatocellular damage from decreased perfusion, whereas high levels of ALP and increased bilirubin have been associated with cholestatic liver injury from an increased central venous pressure [7]. Two HF trials performed in recent past, the EVEREST (Efficacy of Vasopressin Antagonism in Heart Failure Outcome Study with Tolvaptan) and the CHARM (Candesartan in Heart Failure Assessment of Reduction in Mortality and Morbidity) trial suggested that minor abnormalities are common in patients hospitalized for worsening HF with reduced ejection fraction without a history of significant primary liver disease or acute hepatic failure [8].

Typically, a liver specific ALP elevation disproportionate to the rise in aminotransferase suggests a cholestatic disorder. A four-fold elevation of the serum alkaline phosphatase is seen in approximately 75% of patients with chronic cholestasis, both intrahepatic and extrahepatic, whereas lesser elevations are nonspecific, and can occur in a wide range of conditions [9]. In our patient, imaging studies along with biopsy findings correlated with a partial cholestasis picture, but the ALP levels were much higher than expected. To date there is no evidence in the literature that chronic biliary cholestasis with congestive hepatopathy is additive in the ALP levels. There have been a few reports on extremely elevated ALP (greater than 1,000 U/L) in patients with progressive severe HF [10], but these patients typically had worsening renal function on hospital admission.

### Conclusion

A persistently elevated level of ALP often leads to further evaluation. Mild to moderate elevation of ALP in HF patients has been well documented in the literature, but very high levels of ALP are rarely reported. Our patient showed an improvement in the levels of ALP after aggressive diuretic therapy suggesting that the congestive hepatopathy was the most likely cause of highly elevated ALP level. Future clinicians who are managing patients with systolic decompensated HF with concurrent biliary sludge and an elevated ALP greater than what is typically expected should consider congestive hepatopathy as the etiologic source. Therefore, a liver biopsy might not be warranted if the clinical suspicion is low for other pathologies, with normal serological studies and appropriate negative imaging studies.
